# Divergent fifteen-year trends in traditional and cardiometabolic risk factors of cardiovascular diseases in the Seychelles

**DOI:** 10.1186/1475-2840-8-34

**Published:** 2009-06-26

**Authors:** Pascal Bovet, Sarah Romain, Conrad Shamlaye, Shanti Mendis, Roger Darioli, Walter Riesen, Luc Tappy, Fred Paccaud

**Affiliations:** 1 University Institute for Social and Preventive Medicine (IUMSP) and University Hospital Center, Lausanne, Switzerland; 2 Ministry of Health and Social Development, Victoria, Republic of Seychelles; 3 Chronic Disease Prevention and Management, World Health Organization, Geneva, Switzerland; 4 Lipid Laboratory, University Medical Policlinic, University Hospital Center, Lausanne, Switzerland; 5 Institute of Clinical Chemistry and Hematology, Canton Hospital, St Gallen, Switzerland; 6 Department of Physiology, University of Lausanne, Switzerland

## Abstract

**Objective:**

Few studies have assessed secular changes in the levels of cardiovascular risk factors (CV-RF) in populations of low or middle income countries. The systematic collection of a broad set of both traditional and metabolic CV-RF in 1989 and 2004 in the population of the Seychelles islands provides a unique opportunity to examine trends at a fairly early stage of the "diabesity" era in a country in the African region.

**Methods:**

Two examination surveys were conducted in independent random samples of the population aged 25–64 years in 1989 and 2004, attended by respectively 1081 and 1255 participants (participation rates >80%). All results are age-standardized to the WHO standard population.

**Results:**

In 2004 vs. 1989, the levels of the main traditional CV-RF have either decreased, e.g. smoking (17% vs. 30%, p < 0.001), mean blood pressure (127.8/84.8 vs. 130.0/83.4 mmHg, p < 0.05), or only moderately increased, e.g. median LDL-cholesterol (3.58 vs. 3.36 mmol/l, p < 0. 01). In contrast, marked detrimental trends were found for obesity (37% vs. 21%, p < 0.001) and several cardiometabolic CVD-RF, e.g. mean HDL-cholesterol (1.36 vs. 1.40 mmol/l, p < 0.05), median triglycerides (0.80 vs. 0.78 mmol/l, p < 0.01), mean blood glucose (5.89 vs. 5.22 mmol/l, p < 0.001), median insulin (11.6 vs. 8.3 μmol/l, p < 0.001), median HOMA-IR (2.9 vs. 1.8, p < 0.001) and diabetes (9.4% vs. 6.2%, p < 0.001). At age 40–64, the prevalence of elevated total cardiovascular risk tended to decrease (e.g. WHO-ISH risk score ≥10; 11% vs. 13%, ns), whereas the prevalence of the metabolic syndrome (which integrates several cardiometabolic CVD-RF) nearly doubled (36% vs. 20%, p < 0.001). Data on physical activity and on intake of alcohol, fruit and vegetables are also provided. Awareness and treatment rates improved substantially for hypertension and diabetes, but control rates improved for the former only. Median levels of the cardiometabolic CVD-RF increased between 1989 and 2004 within all BMI strata, suggesting that the worsening levels of cardiometabolic CVD-RF in the population were not only related to increasing BMI levels in the interval.

**Conclusion:**

The levels of several traditional CVD-RF improved over time, while marked detrimental trends were observed for obesity, diabetes and several cardiometabolic factors. Thus, in this population, the rapid health transition was characterized by substantial changes in the patterns of CVD-RF. More generally, this analysis suggests the importance of surveillance systems to identify risk factor trends and the need for preventive strategies to promote healthy lifestyles and nutrition.

## Introduction

Cardiovascular disease (CVD) is leading the growing burden of chronic noncommunicable diseases (NCD), including in developing countries [1-3]. The good news is that NCDs, and particularly CVD, are largely preventable [4,5]. Large cohort studies conducted several decades ago have shown that a large proportion of CVD events could be attributed to three "classical" cardiovascular risk factors (traditional CVD RF: tobacco smoking, raised blood pressure, raised blood cholesterol) [6]. Recent evidence from the INTERHEART study suggests that the relative risk of CVD associated with these CVD RF is fairly constant across populations [7], including in Africa [8].

Correspondingly, diet, physical activity and smoking are key determinants of CVD and other NCDs [9]. For example, the expected impact of combining not smoking, moderate alcohol intake, regular physical activity, and high intake of fruit and vegetables is a 80% reduction of both CVD and all-cause mortality [10]. Furthermore, the clustering of several risk factors of metabolic origin, which are associated with unhealthy diet, lack of physical activity and overweight, -e.g. increased triglycerides, decreased HDL-cholesterol, impaired fasting glucose, hyperinsulinemia, diabetes- seems to increase vascular risk [11]. The so-called metabolic syndrome is realized when several of these factors are simultaneously present in the same individual: this specific clustering apparently reflects a profound metabolic dysfunction [12,13]. The frequency of the metabolic syndrome seems to increase alongside the worldwide raising prevalence of obesity.

The monitoring of population levels of the main CVD-RF and related behaviors is the backbone for surveillance of CVD and other NCDs [14,15]. However, reliable information is limited in developing countries. While a number of studies have reported the point prevalence of some CVD-RF, few -if any- have provided population-based trends of both traditional and cardiometabolic CVD-RF in the African region. Such data are however crucial to characterize epidemiological changes at population level [16] in order to provide clues on the driving forces of the epidemic of NCDs and to guide prevention and control strategies.

In this study, we report the 15-year trends of a broad set of both the main traditional CVD-RF and several cardiometabolic CVD-RF in the Seychelles, a rapidly developing country in the African region, based on two comprehensive population-based surveys in 1989 and 2004. Of note, previous studies in this population had documented a high CVD mortality [17], a high prevalence of peripheral atherosclerosis [18] and high levels of traditional CVD-RF [19,20]. The considered time interval may correspond to a fairly early stage of the "diabesity" epidemic in a middle-income country in epidemiological transition.

## Methods

The Republic of Seychelles is a rapidly developing small island state in the Indian Ocean (African region), east of Kenya and north of Mauritius. The population size was 67'000 in 1989 (44% aged ≥25 years) and 84'000 in 2004 (57% aged ≥25 years), which reflects a rapid demographic transition. This underlies the need for age-standardization when comparing results over time. The majority of the population is of African descent. Cardiovascular diseases account for nearly 40% of total mortality [21]. Life expectancy at birth increased from 63 to 69 years in men and from 73 to 76 years in women between 1989 and 2004. The gross domestic product (GDP) per capita rose, in real terms, from $2927 in 1980 to US$ 5239 in 2004, driven by booming tourism, industrial fishing and services. Health care is available with no fee to all inhabitants through a national health system.

### Sampling frame of surveys

Independent population-based examination surveys of CVD-RF were conducted in 1989 [19,22] and in 2004 [20,23]. For both surveys, the sampling frame consisted of a random sex- and age-stratified sample of the population aged 25–64 years. The samples were drawn from the population of the main island in 1989 (which accounts for 90% of the total population) and from the entire population in several islands in 2004. Eligible individuals were selected from an electronic database derived from population censuses carried out in 1987 and 2002, regularly updated thereafter by civil status authorities. In 1989, 1309 individuals we sampled; among them 58 were dead or had emigrated at the time of the survey or could not be traced, and 1081 participated in the survey (86.4%). In 2004, 1632 individuals were sampled; among them 37 were dead or had emigrated at the time of the survey and 32 could not be traced, and 1255 participated in the survey (80.2%). For both surveys, a letter was sent to the eligible participants approximately two weeks prior the appointments to invite them to attend the survey at specified study centers. Other dates for appointments could be arranged and substantial efforts were made to trace the non-participants (e.g. information in the mass media, repeat invitation letters, phone calls, contacts through district administrators, etc). Both surveys were conducted under the auspices of the Ministry of Health, following technical and ethical reviews. Participants gave oral (1989) and written (2004) informed consent.

### Measurement of lifestyle-related and clinical variables

In both surveys, trained officers administered a structured questionnaire on demographic, lifestyle, and other variables to the participants using same or similar questions with regards to the variables considered in this report. Current tobacco use was defined as smoking at least one cigarette every day. Alcohol intake was assessed from a set of questions on drinking frequency and volume for the six main available alcoholic beverages (beer, wine/liquor, spirits and three locally made homebrews), taking advantage that only a limited number of brands and contents were available in the country up to 2004. Questions on alcohol were administered to all participants in 1989 but only to the participants who reported to drink at least once a month in 2004 (hence a slight underestimation of alcohol intake in 2004 vs. 1989). Mean daily ethanol intake was calculated. Definitions of "standard units" and "sensible drinking" vary substantially across countries (e.g.  for definitions around the world). A standard unit most often ranges from 8 to 14 g ethanol per day (or equivalently 10–18 ml ethanol per day) and, in several countries, an intake of ethanol of approximately 1–29 ml/day may correspond to "low risk"; 30–59 ml/day to "moderate risk", and larger intake to "high risk".

Consumption of fruit and vegetables and physical activity was assessed only in 2004 based on the standard STEPS methodology for surveillance of chronic diseases [14,23]. The intake of fruit and vegetables was based on four questions. A daily intake of at least 5 portions of fruit and vegetables is recommended [14]. Physical activity was assessed using the Global Physical Activity Questionnaire (GPAQ), which includes 16 questions assessing the frequency and intensity of physical activity at work, walking to and from places (and not for leisure or work), and physical activity at leisure time [14]. For the calculation, 4 and 8 MET were assigned to the time spent in moderate and vigorous activities, respectively. Light, moderate and high levels of physical activity were defined as <600, 600–2999, and ≥3000 MET-minutes per week, respectively [14].

Measurements of clinical variables were made in 1989 and 2004 using largely similar methods. Weight was measured with calibrated medical electronic scales (Seca) and height was measured using fixed stadiometers. Body mass index (BMI) was calculated as weight divided by squared height (kg/m^2^). Overweight, obesity and extreme obesity were defined for BMI of 25–29, 30–39, and ≥40 kg/m^2^, respectively [24]. BP was measured with a mercury sphygmomanometer using a cuff adapted to the arm circumference. BP was based on the two last of three readings taken at intervals of at least 2 minutes, after the participants had been quiet in the study center for at least 30 minutes and was seated for >10 minutes. Smoking was not allowed in the study centers. We used the target of <140/90 mmHg for "controlled" BP [25].

### Measurement of biochemical variables

In both surveys, fasting blood was collected in the early morning, blood was spun at the study centers, and serum was immediately frozen to -20°C. For both surveys, participants were requested, in their invitation letter, to fast from midnight, except for water.

Except for blood glucose, all analyses were performed at university laboratories in Switzerland. In 1989, total cholesterol (TC) was measured enzymatically (CHOD-PAP method) using reagents from Boehringer (Manheim, Germany) and high density lipoprotein cholesterol (HDL-C) was similarly measured in the supernatant after precipitation of non HDL lipoproteins with phosphotungstate and MgCl^2^. In 2004, blood lipids were measured using a Hitachi 917 instrument and Roche reagents. Day-to-day coefficients of variation (CV) were 2.6% for total cholesterol, 4.1% for HDL-cholesterol, 4.8% for triglycerides in 1989, and 1,8%, 3.6%, and 4.5%, respectively, in 2004. Low-density lipoprotein cholesterol (LDL-C) was calculated with the Friedewald formula. Total cholesterol ≥5.2 mmol/l (200 mg/dl) and ≥6.2 mmol/l (240 mg/dl) defines "borderline high" and "high" levels, respectively [26]. Total cholesterol ≥8.0 mmol/l (≥320 mg/dl) places a person at high total CVD risk [27]. LDL-cholesterol ≥3.4 mmol/l (130 mg/dl) defines a "borderline high" level. HDL-cholesterol <1.0 mmol/l (40 mg/dl) defines a "low" level (i.e. a risk factor) and a value ≥1.5 mmol/l (60 mg/dl) defines a "high" level (i.e. a preventive factor) [26]. Triglycerides ≥1.7 mmol/l (150 mg/dl) define a "borderline-high" level [26].

Fasting blood glucose (FBG) was determined immediately after blood drawing using point-of-care instruments. In 1989, measurements were made on venous blood using a reflectance meter (Reflomat with Hemoglucotest reagent strips, Boerhinger), a validated and frequently used glucometer at the time, which adapts readings to plasma values [28]. In case of elevated FBG and no diabetes history, a second measurement was made 60–120 minutes later and the last measurement was considered. In 2004, measurements were made on venous blood using a Cholestec LDX analyzer (Cholestec, Haywward, USA), a reliable alternative to conventional laboratory devices [29]; the instrument separates blood cells from plasma and measurements are made on plasma. Coefficients of variation for glycemia was 7.5% with Reflomat (1989) and <4% with Cholestec LDX. In case of FBG and no diabetes history, another measurement was carried out within 10 minutes on capillary blood (Ascentia Elite, Bayer) and the mean of the two values was used. The Ascentia Elite partially adjusts capillary blood readings to plasma values [30]. Hence, although different instruments and sequences were used, methods were fairly similar in 1989 and 2004, making a substantial systematic bias in results in 2004 vs. 1989 unlikely. Impaired fasting blood glucose was defined for FBG of 5.6–6.9 mmol/l (100 mg/dl-125 mg/dl) and diabetes for FBG ≥7.0 mmol/l (126 mg/dl), along current recommendations [31].

Fasting serum insulin (FSI) was measured using commercial radio-immuno assay (RIA) kits (1989: charcoal-coated RIA; 2004: LINCO Research Inc, Missouri, USA). Within-run and day-to-day CV were <6% in both 1989 and 2004. The RIA used in 1989 may overestimate insulin concentration in individuals with high concentration of proinsulin or proinsulin splits [32]. HOMA-IR (homeostasis model assessment of insulin resistance) was calculated as [FSI (μU/ml) × FBG (mmol/l)]/22.5] [33] and has been shown to be a reliable estimate of insulin resistance both among diabetic and non diabetic subjects [34,35].

### Cardiovascular disease risk prediction scores

We calculated different scores to estimate an individual's total CVD risk: a) a simple combination of the main traditional CVD-RF (smoking, high BP and raised cholesterol, or the same and diabetes) and b) the CVD risk prediction score for the African region D (WHO/ISH risk score). The WHO/ISH risk score was established by the World Health Organization and the International Society of Hypertension and it indicates the ten-year risk of a fatal or non-fatal cardiovascular event (myocardial infarction or stroke) according to age, sex, BP, smoking status, cholesterol level and diabetes status [36,37]. We restricted all score analyses to persons aged ≥40 years because the WHO/ISH risk score is not designed for younger individuals (the proportion of persons aged 40–64 in the population of Seychelles constitutes nearly half of the population aged 25–64). We defined the metabolic syndrome according to the updated ATP III criteria [38], however substituting obesity for increased waist circumference, as we did not have waist circumference in 1989, and considering that a BMI ≥30 kg/m^2 ^is a criterion used in another definition of the metabolic syndrome [39]. Based on data in 2004, the prevalence of the metabolic syndrome using BMI ≥30 kg/m^2 ^as a criterion slightly underestimated the prevalence of the syndrome using waist as a criterion (35.8% based on BMI ≥30 vs. 39.5% based on increased waist circumference, at age ≥40), and the agreement between the two definitions of the metabolic syndrome was high (kappa was 97% in men and 94% in women, p < 0.001).

### Statistics

All prevalences, means and medians reported in this report are weighted for age, separately for 1989 and 2004, using the new World Health Organization standard population [40]. Results in 2004 vs. 1989 can therefore be directly compared (i.e. differences do not reflect the changing age structure of the populations between 1989 and 2004). Means are displayed for variables normally distributed and medians for variables with skewed distribution. Differences in means, medians and proportions were tested with ANOVA, rank sum test and chi-square test, respectively. Analyses were performed with Stata 8.2 and *P *values less than 0.05 were considered significant.

## Results

### Trends in conventional and cardiometabolic risk factors

Mean age of the participants (upon age standardization) was 42 years (standard deviation: 11) for men and women in both 1989 and 2004. Table 1 shows the trends in BMI, smoking and alcohol intake between 1989 and 2004 as well as the prevalence of categories of physical activity and fruit and vegetable intake in 2004. Mean BMI was higher in women than in men in both surveys and was markedly higher in 2004 than in 1989 in both genders: in 2004, 52% of men and 68% of women were overweight or obese. (BMI ≥25 kg/m^2^). The prevalence of cigarette smoking decreased markedly (from 52% to 31% in men, from 10% to 4% in women). Mean alcohol intake was substantially lower in 2004 than in 1989. Homebrew drinkers had higher ethanol intake from homebrews than drinkers of commercial drinks had from commercial drinks, and these mean alcohol intakes did not change substantially between 1989 and 2004. However, the prevalence of drinkers of homebrews decreased markedly between 1989 and 2004, which contributed to a large decrease in total alcohol intake in 2004 vs. 1989. The prevalence of men and women reporting the recommended amount of ≥5 portions of fruit and vegetables per day (in 2004) was very low. Approximately 80% of men and women were classified as having either moderate or high physical activity. However, the largest part of this reported physical activity was related to physical activity at work and only 41% of men and women reported physical activity during their leisure time.

**Table 1:** Age-standardized trends in body mass index, smoking and alcohol consumption, 1989–2004, and categories of physical activity and fruit and vegetable intake in 2004

	Men			Women			Total		
			
Value	1989	2004	*P*	1989	2004	*P*	1989	2004	*P*
**Body mass index **(kg/m^2^)									
mean (SD)	23.3 (3.7)	25.5 (4.7)	***	25.9 (5.7)	28.3 (6.3)	***	24.6 (5.0)	26.9 (5.7)	***
<18.5	8	4		3	1		5	3	
18.5–24.9	62	39		35	21		49	30	
25.0–29.9	23	33		27	26		25	30	
30.0–39.9	6	22		32	43		19	32	
≥40	1	2		3	9		2	5	
**Daily cigarette smoking**									
Yes	50	36	***	10	4	***	30	17	***
mean cig/day per smoker	12.7	30.8	*	6.9	8.1	ns	11.7	10.1	*
**Alcohol intake **(ml/day)									
mean (SD)	71 (86)	40 (62)	**	9 (29)	4 (13)	**	40 (81)	22 (61)	*
0	25	39		72	82		48	61	
1–29	19	22		20	14		19	18	
30–74	23	24		5	3		14	14	
75–119	14	7		2	1		8	4	
≥120	20	7		1	0		11	4	
All alcoholic beverages									
percent drinkers	75	61	***	30	18	***	52	39	***
mean alcohol per drinker	94.8	65.9	***	30.5	20.4	*	77.5	55.5	***
Commercial alcoholic beverages									
percent drinkers	71	58	***	26	18	***	49	38	***
mean alcohol per drinker	40.1	41.7	ns	16.7	17.7	ns	33.7	36.2	ns
Homebrews									
percent drinkers	37	12	***	8	1	***	23	6	***
mean alcohol per drinker	114.7	135.4	ns	44.3	41.5	ns	104.8	126.1	ns
**Fruit and vegetables (%)**									
<3 portions per day		65			63			64	
3–4 portions per day		33			33			33	
≥5 portions per day		2			4			3	
**Physical activity**									
Physical activity at work									
percent reporting activity^a^		61			62			61	
median MET-min/week		2880			2880			2880	
Walking to places									
percent reporting activity^a^		57			71			64	
median MET-min/week		700			600			600	
Physical activity during leisure time									
percent reporting activity^a^		41			41			41	
median MET-min/week		1200			600			720	
Any physical activity (percent)									
low (<600)		19			17			18	
moderate (600–2999)		36			43			40	
high (>3000)		45			40			42	

Results are presented as percentage (prevalence), mean or median.

ns: not statistically significant; * p < 0.05; ** p < 0.01; *** p < 0.001.

^a ^report physical activity for at least 10 min at a time during on at least one day per week in the specified domain.

Table 2 shows changes in BP, blood lipids, fasting blood glucose, diabetes, insulin and HOMA-IR between 1989 and 2004. Mean BP slightly decreased over time. The prevalence of persons taking antihypertensive treatment increased greatly over time (from 9% to 24%). The prevalence of "hypertension" (BP ≥140/90 mmHg or taking antihypertension treatment) was similar in 1989 and 2004). Mean serum total cholesterol levels, which were substantially lower in men than in women in 1989, increased largely over time in men, but only modestly in women, resulting in almost identical levels in men and in women in 2004. Almost two thirds of adults had elevated or high total cholesterol levels (≥5.2 mmol/l) in 2004. Mean HDL-cholesterol levels were higher in men than in women in 1989. Serum HDL-cholesterol levels were markedly higher in 2004 vs. 1989 in men, consistent with increased prevalence of overweight and decreased alcohol intake during the interval. Mean HDL-cholesterol levels remained virtually unchanged in women. Consequently, mean HDL-cholesterol levels became slightly higher in women than in men in 2004. Median triglyceride levels tended to increase over time (particularly in men). Mean fasting blood glucose levels markedly increased between 1989 and 2004 both in men and women. Approximately one third of men and women had impaired fasting blood glucose levels in 2004 (i.e. FBG between 5.6 and 6.9 mmol/l). The prevalence of diabetes (FBG ≥7.0 mmol/l or glucose lowering treatment) increased by approximately 50% between 1989 and 2004 (overall: 6.1% in 1989 to 9.4% in 2004). Median levels of fasting serum insulin and HOMA-IR (which reflects insulin resistance) markedly increased over time both in men and women.

**Table 2:** Age-standardized trends in blood pressure, blood lipids, blood glucose, diabetes, insulin and HOMA-IR, 1989–2004

	Men			Women			Total		
			
	1989	2004	*P*	1989	2004	*P*	1989	2004	*P*
**BP **(mmHg)									
Systolic (mean)	133 (21)	131 (18)	ns	127 (23)	124 (19)	*	130 (22)	128 (19)	**
Diastolic (mean)	87 (14)	85 (12)	*	83 (14)	81 (13)	ns	845 (14)	83 (12)	*
<120/80	21	19		34	40		28	30	
120–139/80–89	35	42		33	35		33	38	
140–159/90–99	25	25		21	16		23	21	
160–169/100–109	17	12		10	8		14	10	
≥180/110	2	2		2	1		2	1	
≥140/90 or BP treatment	45	44		35	36		40	40	
Medication for high BP									
Yes	7	21	***	11	26	***	9	24	***
**Total cholesterol **(mmol/l)									
Mean	5.07 (1.10)	5.42 (1.26)	***	5.33 (1.18)	5.40 (1.20)	ns	5.20 (1.14)	5.41 (1.26)	***
<5.2	60	50		48	49		55	48	
5.2–6.1	30	28		29	28		29	29	
6.2–7.9	9	20		20	20		15	20	
≥8.0	1	2		3	3		2	3	
**HDL cholesterol **(mmol/l)									
Mean	1.43 (0.51)	1.35 (0.53)	*	1.38 (0.36)	1.36 (0.41)	ns	1.40 (0.44)	1.36 (0.47)	*
<1.0	16	26		12	16		14	21	
1.0–1.4	49	43		57	50		53	47	
≥1.5	35	31		31	34		33	32	
**Triglycerides **(mmol/l)									
Median	0.81	0.90	**	0.75	0.80	ns	0.78	0.80	**
>1.7	12	18		6	8		9	13	
**LDL cholesterol **(mmol/l)									
Mean	3.17 (1.09)	3.54 (1.25)	***	3.55 (1.10)	3.61 (1.16)	ns	3.36 (1.11)	3.58 (1.20)	***
≥3.4	37	50		52	53		44	52	
**Fasting blood glucose **(mmol/l)									
Mean	5.21 (1.72)	6.03 (2.13)	***	5.24 (2.19)	5.75 (1.81)	***	5.22 (1.97)	5.89	***
<5.6	76	60		78	73		77	67	
5.6–6.9	18	31		16	19		17	25	
≥7.0	5.9	9.2		5.8	7.9		5.8	8.5	
≥7.0 or diabetes treatment	6.2	9.6	***	6.2	9.1	***	6.2	9.4	***
Medication for diabetes									
Yes	1.4	4.3	*	2.0	5.8	*	1.7	5.0	**
**Fasting insulin **(μmol/ml)									
Median	6.8	10.5	***	9.3	12.7	***	8.3	11.6	***
**HOMA-IR**									
Median	1.6	2.7	***	2.0	3.1	**	1.8	2.9	***
≥4.0 (upper quartile)	8	27	***	13	33	***	11	30	***

Results are presented as percentage (prevalence), mean or median.

ns: not statistically significant; * p < 0.05; ** p < 0.01; *** p < 0.001.

**Figure 1:**
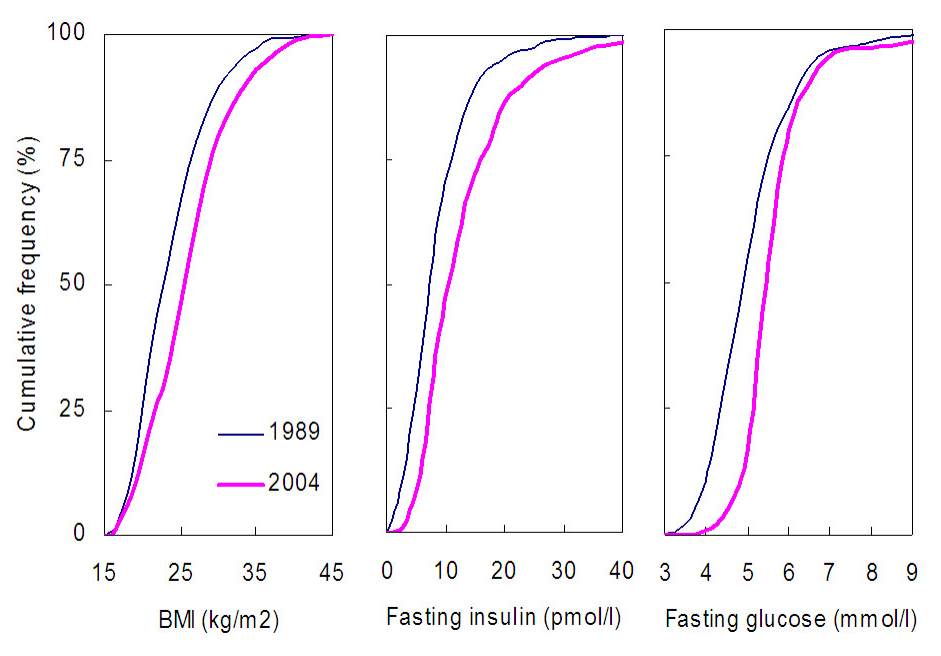
**Age-standardized cumulative distribution of body mass index, fasting insulin and fasting glucose in 1989 and 2004**.

The entire age-adjusted distributions of BMI, fasting insulin and fasting glucose markedly shifted toward higher values in 2004 vs. 1989 (Figure 1). There was a trend towards a larger shift in the upper range than lower range for BMI and insulin. For glucose, the shift seemed to be larger for the lower than higher values of the distribution. Data are based on all participants, including those treated. Of note, very few diabetic participants were treated with insulin, and oral glucose lowering medications are expected to only minimally lower glucose (e.g. by ~1–2 mmol/l) as compared to non-treated values. The fairly similar shifts for the three cardiometabolic conditions suggest that these three factors are strongly inter-related.

**Figure 2:**
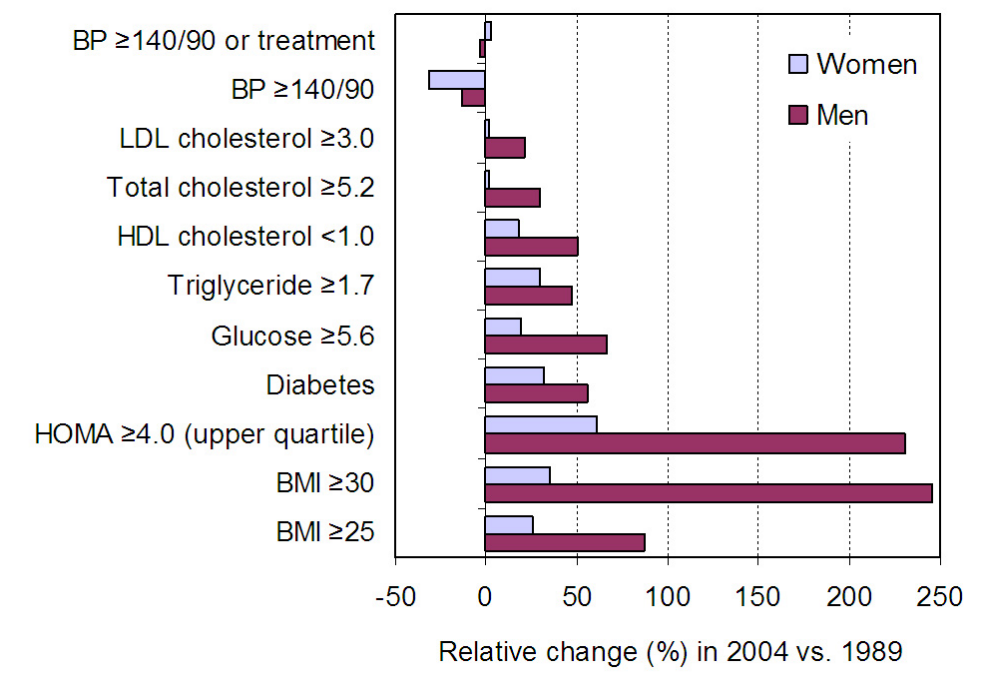
**Age-standardized proportionate changes in the prevalence of selected cardiovascular risk factors in 1989 and 2004**.

The proportionate changes in prevalences, in 2004 vs. 1989, were markedly different for the main traditional CVD-RF vs. the cardiometabolic CVD-RF (Figure 2). For the main three traditional CVD-RF, the prevalence either decreased (smoking), did not substantially change (hypertension, i.e. BP ≥140/90 or treatment), or slightly increased (elevated cholesterol). In contrast, the proportionate changes over time were largely unfavorable for overweight and obesity, and for most of the considered metabolic CVD-RF (low HDL-cholesterol, high triglyceride, abnormal fasting glucose, upper quartile of HOMA-IR). A larger relative increase in men than in women for most of these obesity-related cardiometabolic CVD-RF is consistent with the proportionately larger increase in overweight/obesity in men than in women. Of note, the absolute increase in mean BMI, between 1989 and 2004, was similar in men (+2.2 kg/m^2^) and in women (+2.4 kg/m^2^). However, because mean BMI was substantially lower in men than in women in 1989 (with more women than men being already overweight or obese in 1989), the proportionate increase in the prevalence of overweight and obesity, between 1989 and 2004, was greater in men than in women. Of note, mean height changed by less than 0.2 cm between 1989 and 2004 both in men and women aged 25–64 (P > 0.2), hence changes in BMI over time almost entirely reflects increasing weight.

### Trends in CVD prediction scores based on traditional and cardiometabolic risk factors

The age-standardized prevalence of categories with several main traditional CV-RF among men and women aged 40–64 years tended to decrease (although not significantly) between 1989 and 2004 (Table 3). Similarly, the prevalence of men and women with elevated total CVD risk, based on the WHO/ISH risk score, tended to decrease both in men and in women (except for women with a score ≥20). In contrast, the prevalence of the metabolic syndrome increased greatly in men (13% to 32%, P < 0.001) and in women (26% to 40%, P < 0.001).

**Table 3:** Age-standardized trends in the prevalence of cardiovascular risk prediction scores based on traditional and cardiometabolic risk factors, 1989–2004

	Men			Women			Total		
			
	1989	2004	*P*	1989	2004	*P*	1989	2004	*P*
≥1 of 3 traditional risk factors	93	90	ns	83	81	ns	88	85	ns
≥2 of 3 traditional risk factors	55	47	ns	43	36	*	49	42	**
3 of 3 traditional risk factors	10	8	ns	2	1	*	6	5	ns
									
≥1 of 4 traditional risk factors	93	90	ns	84	82	ns	88	86	ns
≥2 of 4 traditional risk factors	57	51	ns	43	41	ns	50	46	ns
≥3 of 4 traditional risk factors	14	17	ns	10	10	ns	12	13	ns
4 of 4 traditional risk factors	3	2	ns	1	0	ns	2	1	ns
									
WHO/ISH score AFRO ≥ 10	14	11	ns	13	11	ns	13	11	ns
WHO/ISH score AFRO ≥ 20	7	5	ns	4	5	ns	6	5	ns
									
Metabolic syndrome	13	32	***	26	40	***	20	36	***

ns: not statistically significant; * p < 0.05; ** p < 0.01; *** p < 0.001.

DM: diabetes mellitus. WHO/ISH score AFRO: CVD risk prediction score for the African region D.

Major risk factors (3): blood pressure ≥140/90 mmHg or treatment; daily smoking; total cholesterol ≥5.2 mmol/l.

Major risk factors (4): same as above + diabetes (fasting blood glucose ≥7.0 mmol/l or treatment).

Metabolic syndrome using ATP III criteria modified with BMI ≥30 kg/m^2 ^instead of elevated waist circumference.

### Awareness, treatment and control rates for high blood pressure, diabetes and dyslipidemia

Awareness, treatment and control rates increased markedly over time for high BP (Table 4). For diabetes, awareness increased markedly, and almost all persons aware of diabetes were treated in 2004, but the control rates among treated persons remained low. For high cholesterol (data available only in 2004), awareness and treatment remained low in 1989 and 2004 but control was high among the few persons treated (reflecting the high efficacy of atorvastatin, which was the lipid-lowering medication used most often in the Seychelles in 2004).

**Table 4:** Age-standardized trends (in %) in awareness, treatment and control of high blood pressure, diabetes and hypercholesterolemia, 1989–2004

	Men			Women			All		
			
	Aware among all with condition	Treated among aware	Controlled among treated	Aware among all with HBP	Treated among aware	Controlled among treated	Aware among all with HBP	Treated among aware	Controlled among treated
High blood pressure (BP ≥140/90 mmHg or treated)									
1989	36	41	9	49	63	16	41	52	14
2004	55	89	24	75	96	42	64	93	34
*P*	***	***	***	***	***	***	***	***	***
Diabetes (fasting blood glucose ≥7.0 mmol/l or treated)									
1989	36	64	28	44	75	24	40	70	26
2004	46	97	15	64	100	26	54	98	21
*P*	ns	**	ns	*	**	ns	*	***	ns
High total cholesterol (≥6.2 mmol/l or treated)									
1989	-	-	-	-	-	-	-	-	-
2004	9	24	78	11	28	42	10	26	57

ns: not statistically significant; * p < 0.05; ** p < 0.01; *** p < 0.001.

Proportions (in %) are from total for "aware"; from aware for "treated", and from treated for "controlled".

Cut off values for "Controlled" are BP <140/90 mmHg for "high blood pressure", fasting blood glucose <7.0 mmol/l for "diabetes", and cholesterol <6.2 mmol/l for "high total cholesterol".

### Trends in traditional and cardiometabolic risk factors within BMI categories

Stratified analysis of CVD-RF levels within BMI categories allows determining whether the secular changes in traditional and cardiometabolic CVD-RF in 2004 vs. 1989 were independent of the increase in BMI in the interval (Table 5). All the considered traditional and metabolic CVD-RF increased, as expected, across increasing categories of BMI. Within the same BMI categories, median systolic and diastolic BP decreased over time (irrespective of antihypertension medication) but the median levels of several metabolic CVD-RF (particularly fasting blood glucose and insulin) substantially increased over time, suggesting a role of factors other than BMI to account for the increasing levels of the cardiometabolic CVD-RF in 2004 vs. 1989.

**Table 5:** Age-standardized trends in median levels of traditional and cardiometabolic risk factors according to body mass index

	BMI <25			BMI 25–29			BMI ≥30		
			
	1989	2004	*P*	1989	2004	*P*	1989	2004	*P*
Systolic BP (mmHg)	122	120	*	132	127	***	138	131	***
Diastolic BP (mmHg)	80	79	*	89	84	***	90	86	***
Systolic BP, not treated (mmHg)	120	118	***	130	122	***	134	126	***
Diastolic BP, not treated (mmHg)	80	77	***	87	81	***	88	84	***
LDL-cholesterol (mmol/l)	2.98	3.23	**	3.66	3.61	ns	3.86	3.67	ns
HDL-cholesterol (mmol/l)	1.39	1.48	*	1.28	1.19	ns	1.24	1.19	ns
Triglyceride (mmol(l)	0.71	0.73	ns	0.93	0.94	ns	0.94	0.98	ns
Glucose (mmol/l)	4.6	5.2	***	5.0	5.5	***	5.2	5.6	***
Insulin (μmol/l)	6.9	8.7	***	9.7	12.8	***	13.3	16.9	***

All data are expressed as median values.

ns: not statistically significant; * p < 0.05; ** p < 0.001; *** p < 0.001.

## Discussion

The major finding of this study is that the population levels of the main traditional CVD-RF either decreased (smoking, high blood pressure) or only slightly increased (elevated total cholesterol) during the considered 15-year interval, while the levels of several cardiometabolic CVD-RF (diabetes, high triglyceride, low HDL-cholesterol, and high HOMA-IR) markedly increased in a country in rapid health transition in the African region. Correspondingly, the prevalence of persons with elevated total CVD risk (using a CVD risk prediction score largely based on the main traditional CVD-RF) tended to decrease over time, whereas the prevalence of the metabolic syndrome doubled. These findings further suggest that the secular increase in adiposity is driving secular changes in the cardiometabolic risk profile in this population.

Several observations can be made in relation to the rather favorable secular trends in several main traditional CVD-RF. The large decrease in the prevalence of tobacco use reflects sustained tobacco control programs during the past decade in the Seychelles, including a ban on tobacco advertisement, increasingly high taxes on cigarettes, and continuous education programs in the mass media [41]. The slight secular downward trend in systolic/diastolic BP is less easy to explain. While treatment and control rates for hypertension markedly increased over time, and alcohol intake decreased, the prevalence of several other determinants of high BP increased, e.g. overweight, insulin resistance and, possibly, sedentary habits. A separate study indicated moderate salt consumption in the Seychelles (~5 grams per day) [42], consistent with a staple diet based on fish and rice. Hence, protective, unaccounted factors may override the impact of rising determinants of BP.

The slight increase in total cholesterol and LDL-cholesterol may relate to a shift from the traditional diet largely made of rice and fish to increased consumption of meat and processed foods. This deterioration in blood lipids levels in the population is fairly small compared to other developing countries [43]: this may relate to the already fairly high cholesterol levels in the Seychelles in 1989 and, perhaps, a shift toward an increasing consumption of vegetable oils low in saturated fats (e.g. corn oil) and a decreasing consumption of palm oil (rich in saturated fats), despite slightly higher prices of the former. This might be attributable to health education programs in the Seychelles, which have repeatedly emphasized on this nutritional issue.

The largest secular change in CVD-RF was observed for overweight and obesity. This is consistent with marked socio-economic changes in the recent decades in the Seychelles, including increased purchasing power (the GDP per capita doubled in 2004 vs. 1989), larger caloric intake, and increasing sedentary behaviors. For examples, calorie availability per capita increased from 1800 to 2300 over the past 20 years [44] and the local production of carbonated soft drinks has tripled in the past 25 years (figures from Seychelles Breweries Ltd). During the same period, the numbers of private cars and passengers transported by buses have doubled (figures from the Licensing Authority and from the Seychelles Public Transport Company).

The association between adiposity, insulin resistance and metabolic CVD-RF are well known [12,13]. We previously showed that insulin resistance was associated with serum triglycerides (directly) and HDL-cholesterol (inversely) in the population of Seychelles [45]. We also described, in this population, a high prevalence of the metabolic syndrome in 2004, irrespective of the definition used [46]; a strong relationship between insulin resistance and BMI, independent of blood glucose impairment [47]; and an association between pre-diabetes and increased artery intima-media thickness [48]. More generally, it is well recognized that the increasing prevalence of obesity is a main driving force behind the rising prevalence of several cardiometabolic CVD-RF, including insulin resistance and diabetes, in populations worldwide [11,49,50]. In our study, the prevalence of overweight and obesity increased proportionately more in men than in women, and so did the levels of several cardiometabolic CVD-RF (although the secular increase in mean BMI was similar, in absolute values, in both genders). This suggests that an increase in the population levels of cardiometabolic CVD-RF may be more sensitive to BMI changes at intermediary vs. high levels. Interestingly, stratified analysis suggested that factors other than adiposity (as measured by BMI, in our study) also accounted for the increasing levels of the cardiometabolic CVD-RF over time.

What is the significance of our observed divergent secular trends in traditional vs. cardiometabolic CVD-RF in the population? Based on a review of all death certificates, the age-adjusted mortality rates for stroke and myocardial infarction (per 100'000 total population) were fairly high by international standards, e.g. 92/69 in men/women for stroke and 64/28 for myocardial infarction in 2002–2005 [17]. However, the rates in 2002–2005 were ~15–30% lower than in 1989–1992 [17]. These downward CVD mortality trends are consistent with decreasing levels of several traditional CVD-RF and decreasing prevalence of high total CVD risk (based on CVD risk prediction scores). This also means that the detrimental trends in cardiometabolic CVD-RF had little detrimental impact on CVD mortality. This may relate to several factors. First, we previously showed that the slope of the relationship between BP and BMI had decreased in 2004 vs. 1989, irrespective of anti-hypertension treatment [51], suggesting a secular decreasing impact of BMI on BP. Similarly, we found that BP decreased during the past decade among children despite a marked increase in the prevalence of overweight [52]. A decreased impact of adiposity over time has also been observed in other populations: both the relative risk of CVD associated with obesity and the mean levels of traditional CVD-RF within BMI strata decreased over time (except for diabetes) in the USA between 1970 and 2000 [53,54].

The discrepancy between the favorable trends in both CVD mortality [17] and predicted CVD mortality (based on the WHO/ISH risk score in this study), on one hand, and the markedly unfavorable trends in cardiometabolic CVD-RF, on the other hand, seems to dismiss a large impact of the cardiometabolic CVD-RF (and insulin resistance) on CVD outcomes, at a population level. There is ongoing controversy on the value of the metabolic syndrome as a physiopathological and clinical concept able to enhance our understanding and our management of CVD risk in patients and in populations. It is not yet clear whether the metabolic syndrome improves the prediction of CVD risk as compared to traditional CVD-RF [55]; if it predicts CVD incidence better than its individual components [55-58]; or even if it performs better than fasting blood glucose for predicting diabetes [58]. However, a time lag may be necessary before this metabolic wave translates into detrimental cardiovascular outcomes in the population. This could explain why the decline in CVD rates only recently flattened (among young adults) in western countries, despite decades of increasing prevalence of obesity [59]. A time lag had also been suggested for the effect of traditional CVD-RF on the incidence of CVD [60]. Alternatively, a detrimental impact related to the increased prevalence of several cardiometabolic CVD-RF and, in particular, increasing insulin resistance in the population might be offset, in our study, by the favorable trends in several major traditional CVD-RF.

These uncertainties regarding the actual impact of obesity and the metabolic syndrome on the incidence of CVD should not lead to overlook the fully acknowledged link between obesity and diabetes. The tide of diabesity is worrying when considering the continuously poor control rates among treated diabetic persons -in contrast to control of hypertension or dyslipidemia. This finding points to the known difficulty to control cardiometabolic conditions because of insulin resistance [61], a situation that should likely result in an increased burden of diabetes-related complications. The unfavorable impact of the rising levels of cardiometabolic CVD-RF on the burden of diabetes-related diseases is illustrated, in the Seychelles, by the increasing proportion of diabetic patients in chronic hemodialysis (approximately a third of all patients under chronic hemodialysis in 2004) and the increasing incidence of diabetes-related amputations recorded at hospital.

There are a few limitations in the study. Laboratory methods were similar, but not identical, in 1989 and 2004, which is inevitable since laboratory methods and instruments change over time. However, except for blood glucose, all biochemical analyses were done at university laboratories using best practices at their time. Furthermore, the consistency in i) the observed secular detrimental changes for all the considered cardiometabolic CVD-RF (e.g. HDL-cholesterol, triglyceride, glucose, insulin) and ii) the good concordance between the proportionate changes in the prevalence of obesity and the proportionate changes in the mean levels of the considered cardiometabolic CVD-RF in men vs. women, suggests coherence in the results. From an epidemiological perspective, one must refrain to equate the category of "high BP or treatment" (BP ≥140/90 mmHg or antihypertensive treatment) to "hypertension", since BP measurements based on only one occasion (like in our study) can substantially overestimate the true prevalence of hypertension [62]. The same remark applies, to a lesser extent, to "elevated blood glucose or treatment" vs. "diabetes". Strong points of the study include the fairly large sample sizes, the truly random selection of the participants, the large participation rates, the comprehensive assessment of both traditional and cardiometabolic CVD-RF, the largely similar methods used in the two surveys and, most importantly, the availability of such data in a country in the African region as early as in 1989 (i.e. at a fairly early stage of the diabesity era).

Our findings have several implications for public health. Overall, the rather favorable secular trends in several main traditional CVD-RF are consistent with the downward trends in age-standardized CVD mortality in this population [17]. This is consistent with downward age-specific trends observed in the western countries [63] and -to a lesser extent- in some developing countries as well [64]. The decreasing smoking prevalence and the improved treatment rates for hypertension in Seychelles are likely related to the national multipronged program against chronic diseases since the early 1990s [41,65]. However, the levels of these major traditional CVD-RF remain high in the population. There is therefore a continued need for public health interventions to further reduce levels of smoking, BP and blood cholesterol in the population [66,67] and for strengthening health services for improved health care of high-risk individuals [68]. On the other hand, the prevalence of obesity and related cardiometabolic CVD-RF have dramatically increased during the past 15 years, which may stem from global social and economic changes that fuel unhealthy nutrition and physical inactivity [69]. The significance of these epidemiological changes on the incidence of CVD cannot be evaluated yet in our data – and further studies will be needed to address this question-, but their impact on the prevalence of diabetes and its complications is already visible. This latter observation is the main reason to take the upward trends in obesity and diabetes in developing countries as a major public health challenge [70]. Since individual-centered approaches for weight prevention and control have limited impact [71], emphasis should be given to policy and structural interventions that promote regular physical activity and healthy nutrition in the entire population [50,72-76]. Finally, our data emphasize the importance of reliable and standardized information systems to identify the changing epidemiological situation and to guide, monitor and evaluate the clinical and public health responses.

## Competing interests

The authors declare to have no competing interests. The views expressed in this paper are solely the responsibility of the named authors and do not necessarily reflect the views of the institution(s) to which they are affiliated.

## Authors' contributions

PB was the principal investigator of both surveys and led the data analysis and the write up of the manuscript; CS and FP participated to the study design of both surveys and reviewed the manuscript; RD, WR and LT performed most of the biochemical analyses and reviewed the manuscript; SR and SM participated to the interpretation of the data and reviewed the manuscript. All authors read and approved the final manuscript.
